# Supranutritional phytase and dietary phosphorus effects on White Leghorns: I. Performance and egg traits

**DOI:** 10.1016/j.psj.2026.107626

**Published:** 2026-08-14

**Authors:** R.M. Horn, S.L. Vieira, D.D.B. Maria, Y.J. Olabarriaga, W.E. Altevogt, L.A.L. da Cunha, R.F.B. Leiskosky

**Affiliations:** Department of Animal Science, Federal University of Rio Grande do Sul, Av. Bento Gonçalves, 7712, Porto Alegre, RS, 91540-000, Brazil

**Keywords:** Laying hen, Phytase, Phosphorus, Egg traits, Nutrient digestibility

## Abstract

This study evaluated the effects of supranutritional phytase supplementation (4,000 FYT/kg) in feeds having varying dietary phosphorus (P) on performance, egg traits, and ileal digestibility of White Leghorns (Bovans White) hens from 26 to 57 wk of age. A total of 324 hens were assigned to a 2 × 6 factorial: feeds with or without phytase vs. six graded increases of dietary P (avP = 0.09 to 0.44 % equivalent to 0.07 to 0.31 % digestible P). Responses were evaluated over eight 28 d periods; ileal digestibility was determined at the end of the trial. Significant phytase × avP interactions were observed for BW gain (BWG), feed conversion ratio (FCR; kg/kg egg), egg production, and the digestibility of DM, energy, and CP (P < 0.05). Without phytase, BWG increased linearly with increasing avP, whereas FCR (kg/kg) and egg production showed quadratic responses, with optimum values at 0.33 and 0.37 % avP, respectively. In phytase-supplemented hens, BWG, FCR (kg/kg egg), egg production, and energy digestibility responded linearly, whereas DM and CP digestibility showed quadratic responses. Phytase supplementation increased BWG (+16.3 %), feed intake (FI; +1.7 %), egg production (+2.0 %), total eggs (+4 eggs/hen), egg weight (+0.5 g), eggshell proportion (+0.19 %), shell breaking strength (+0.17 kgf), shell thickness (+6 μm), and ileal P digestibility (+23.6 %), while improving FCR (kg/kg) and reducing yolk percentage (P < 0.05). Independently of phytase, FI, eggshell proportion, and shell thickness increased linearly with increasing avP, whereas egg weight was maximized at 0.30 % avP. Feed conversion ratio (kg feed/dozen eggs) responded quadratically to avP, with an estimated optimum at 0.34 % avP. Calcium digestibility decreased linearly as avP increased, whereas P digestibility was unaffected by avP (P > 0.05). In conclusion, supranutritional phytase supplementation modified the response of laying hens to dietary P, reducing the avP concentration required to sustain productive performance from 0.33 to 0.37 % to 0.23–0.27 %.

## Introduction

Phosphorus (P), the second most abundant mineral in the animal body, is indispensable for skeletal development. It also plays essential roles in ATP-dependent energy metabolism and the maintenance of acid–base and osmotic homeostasis (Suttle, 2022). Phosphorus deficiency can impair feed intake (FI), thereby compromising egg production (Gordon and Roland, 1998; Nie et al., 2018; Suttle, 2022). Conversely, excessive dietary P may disrupt calcium (Ca) metabolism, reducing its deposition in the eggshell and ultimately compromising shell quality (Roland and Farmer, 1986). Consequently, optimizing dietary P supply is critical for maximizing productivity while maintaining eggshell quality.

Corn and soybean meal (SBM), as well as other plant feedstuffs, contain most of their P in the form of phytate, which is largely unavailable to poultry in the absence of exogenous phytase (Ravindran et al., 1995). This leads to a high proportion of dietary P being excreted rather than retained, increasing feed costs and contributing to P accumulation in agricultural soils following land application of poultry manure, thereby reducing the economic and environmental sustainability of poultry production (Jing et al., 2021; Knowlton et al., 2004; Rutherfurd et al., 2004). Exogenous phytase has become an effective nutritional strategy to hydrolyze phytate, increasing P availability and reducing dependence on inorganic phosphate supplementation.

Despite extensive research demonstrating the efficacy of phytase in broilers, comparatively fewer studies have evaluated its responses in laying hens. Unlike broilers, laying hens consume feeds containing substantially higher Ca concentrations, at wider Ca:P ratios, to sustain continuous eggshell mineralization. These dietary conditions promote the rapid formation of insoluble Ca–phytate complexes, reducing phytate solubility and limiting phytase access to its substrate (Selle and Ravindran, 2007). The unique mineral metabolism of laying hens, characterized by continuous medullary bone turnover and tightly regulated Ca and P homeostasis, may alter phytate utilization and responses to phytase compared with broilers (Bar, 2009).

The prolonged productive lifespan of modern laying hens has further increased concerns regarding the adequacy of current dietary P recommendations. Daily mobilization of medullary bone for eggshell formation releases both Ca and P into circulation, providing an endogenous source of P that may influence dietary requirements (Bar, 2009). Previous studies have estimated non-phytate P requirements between 0.22 and 0.35 % (Gordon and Roland, 1998; Granghelli and Persia, 2022; Keshavarz, 2000; Rodehutscord et al., 2023). However, phytase responses have varied considerably among studies because of differences in hen age, dietary composition, phytase inclusion level, and experimental duration. Furthermore, most available recommendations are based on relatively short-term studies and may not adequately reflect the genetic progress achieved in egg production persistency and egg mass.

Commercial phytase has traditionally been included at approximately 500 FYT/kg in poultry feeds to improve phytate P utilization and reduce the need for inorganic phosphate supplementation (Cowieson et al., 2011; Dersjant-Li et al., 2015). However, under the high-Ca conditions characteristic of commercial layer feeds, conventional phytase doses may not fully overcome the limitations imposed by rapid Ca–phytate complex formation (Ma et al., 2019). Increasing phytase inclusion to supranutritional levels may accelerate phytate hydrolysis before extensive complex formation occurs, thereby maximizing phytate degradation and enhancing the release of P as well as other nutrients associated with the phytate molecule (Cowieson et al., 2011). Indeed, recent studies have evaluated phytase supplementation at both conventional (Jlali et al., 2023; Moura et al., 2023; Musilova et al., 2017) and supranutritional inclusion levels (Pirzado et al., 2024; Ren et al., 2023). In addition to improving P utilization, high phytase supplementation may increase the availability of trace minerals, amino acids, and energy, thereby improving productive performance and feed efficiency, as previously demonstrated in broilers (Feijó et al., 2023; Maria et al., 2024).

Therefore, the objective of the present study was to evaluate the effects of a wide range of dietary available P concentrations, from the lowest level achievable without inorganic phosphate supplementation to current commercial levels, with or without supplementation of a supranutritional phytase dose (4,000 FYT/kg). Responses were evaluated in terms of productive performance, egg quality traits, and the ileal digestibility of P, Ca, DM, crude protein (CP), and gross energy (GE) in Bovans White laying hens from 26 to 57 wk of age. It was hypothesized that supplementation with 4,000 FYT/kg phytase would maximize phytate degradation under the high dietary Ca concentrations and wide Ca:P ratios characteristic of commercial layer feeds, thereby maintaining productive performance and eggshell quality while improving ileal P digestibility.

## Materials and methods

All procedures utilized in the present study were approved by the Ethics and Research Committee of the Federal University of Rio Grande do Sul, Porto Alegre, RS, Brazil (approval number 46471).

### Birds husbandry and dietary treatments

A total of 324 Bovans White pullets originating from a commercial pullet farm (Mercoaves Comércio de Aves Ltda, Bom Princípio, RS, Brazil) were individually placed in cages (0.33 m length × 0.46 m width × 0.40 m height) at 13 wks of age. Photoperiodic stimulation started at arrival with a 13L:11D cycle, followed by weekly increases of 30 min until 16L:8D was reached at 19 wks of age and maintained until the end of the trial. Ambient temperature targeted bird comfort and followed Bovan breeders’ recommendations (Hendrix, 2020).

At 26 wks of age, hens were randomly assigned into a 2 × 6 factorial arrangement of feeds not supplemented or with 4,000 FYT/kg of phytase and six dietary P levels (avP=0.09, 0.16, 0.23, 0.30, 0.37, and 0.44 %, which corresponded to 0.07, 0.12, 0.17, 0.22, 0.26 and 0.31 % digestible P (dP)) in a total of 12 treatments. For feed formulation purposes, dP values were assigned according to Rodehutscord (2013). Performance and egg production were evaluated in eight 28 d periods (26–29, 30–33, 34–37, 38–41, 42–45, 46–49, 50–53, and 54–57 wk). Each dietary treatment was replicated 27 times, with one individually caged hen as the replicated experimental unit. The lowest available phosphorus (avP) treatment (0.09 % avP; 0.07 % dP) was formulated without supplemental inorganic phosphate. In order to formulate the additional treatments with higher P, dicalcium phosphate (24 % Ca and 19 % P) was progressively added to achieve levels approaching those commonly used in commercial layer feeds (0.44 % avP; 0.31 % dP). Each dietary treatment was manufactured once per experimental period in approximately 140 kg batches, with samples collected and stored at −20°C for further analyses.

Phytase was supplied as a commercial product (HiPhorius™, 40,000 FYT/g; Novonesis A/S, Bagsvaerd, Denmark) and incorporated into the feeds at 100 g/ton, corresponding to 4,000 FYT/kg (4,186 ± 432 FYT/kg analyzed). This is a fourth-generation 6-phytase from *Citrobacter braakii* expressed in *Aspergillus oryzae* and it is characterized by high thermostability and optimized activity across the pH conditions of the gastrointestinal tract. The phytase inclusion level exceeded the commercial recommendation to promote extensive and rapid degradation of phytate in corn and SBM, thereby maximizing P release. The additive was added without assigning nutritional values for P or Ca.

Feed formulations followed commercial standards with sequential corn–soybean meal feeds: Grower II (13–16 wks), Pre-Lay (17–20 wks), Layer I (21–25 wks, pre-experimental phase; 26-37 wks, Table 1), and Layer II (38–57 wks). The Grower II feed contained 2,750 kcal/kg AMEn, 17.0 % CP, 1.5 % Ca, and 0.45 % avP (0.32 % dP), whereas the Pre-Lay feed provided 2,800 kcal/kg AMEn, 17.3 % CP, 2.2 % Ca, and 0.50 % avP (0.35 % dP). The Layer I feed contained 2,860 kcal/kg AMEn, 17.4 % CP and 4.1 % Ca (3.77 % ± 0.33), while the Layer II feed provided 2,860 kcal/kg AMEn, 17.0 % CP and 4.2 % Ca (3.84 ± 0.13) (Table 1).

**Table 1:** Ingredient and nutrient composition of feeds having supplemental phytase and graded dietary P increases.

Ingredients, % as-is	Layer I (26 to 37)						Layer II (38 to 57)					
	0.09	0.16	0.23	0.30	0.37	0.44	0.09	0.16	0.23	0.30	0.37	0.44
Corn	55.0	54.8	54.6	54.4	54.3	54.1	55.7	55.4	55.3	55.1	54.9	54.7
Soybean meal, 46 %	28.7	28.7	28.7	28.8	28.8	28.8	27.6	27.7	27.7	27.7	27.8	27.8
Soybean oil	3.1	3.1	3.2	3.3	3.3	3.4	3.06	3.12	3.19	3.26	3.32	3.39
Dicalcium phosphate	-	0.40	0.80	1.10	1.50	1.8	-	0.34	0.67	1.11	1.44	1.77
Limestone	12.1	11.9	11.6	11.3	11.0	10.8	12.4	12.2	11.9	11.6	11.3	11.1
Salt	0.34						0.37					
Methionine analogue^1^	0.29						0.26					
L-Lysine HCL	0.01						0.01					
L-Threonine	0.03						0.11					
Vitamin and minerals^2^	0.35						0.35					
Choline chloride	0.12						0.13					
Total	100.00						100.00					
AMEn, kcal/kg	2,860						2,860					
Crude protein	17.35						17.00					
Ca	4.1						4.2					
AvP	0.09	0.16	0.23	0.30	0.37	0.44	0.09	0.16	0.23	0.30	0.37	0.44
Dig. P	0.07	0.12	0.17	0.22	0.26	0.31	0.07	0.12	0.17	0.22	0.26	0.31
NPP	0.08	0.14	0.21	0.28	0.34	0.41	0.08	0.14	0.21	0.28	0.34	0.41
Total P^4^	0.29	0.35	0.42	0.49	0.55	0.62	0.29	0.35	0.42	0.49	0.55	0.62
Na	0.17						0.18					
d Lys	0.85						0.83					
d Met	0.50						0.47					
d TSAA	0.76						0.72					
d Trp	0.149						0.19					
d Thr	0.60						0.66					
d Val	0.75						0.73					
Choline, mg/kg	1,700						1,700					
Phytate^5^	0.21						0.20					
Phytase, FYT/kg^6^	4,208±482						4,164±308					

1 Novus (DL-2‑hydroxy-(4-methylthio) butanoic acid).

2 Composition per kg of feed: vitamin A, 14,875 IU; vitamin D₃, 5,775 IU; vitamin E, 35 IU; vitamin K₃, 5.25 mg; vitamin B₁, 3.5 mg; vitamin B₂, 8.05 mg; vitamin B₆, 5.25 mg; pantothenic acid, 21 mg; vitamin B₁₂, 43.75 µg; nicotinic acid, 70 mg; folic acid, 1.40 mg; biotin, 0.35 mg; Se, 0.455 mg; Fe, 78.75 mg; Mn, 140 mg; Zn, 96.25 mg; I, 1.75 mg; Cu, 11.38 mg; butylated hydroxytoluene, 100.1 mg; Rovabio Advance (Adisseo France S.A.S., Antony, France), 500 mg.

3 Analyzed P with and without phytase: Layer 1, 0.34 ± 0.02, 0.41 ± 0.02, 0.49 ± 0.02, 0.55 ± 0.02, 0.62 ± 0.03 and 0.69 ± 0.04%; Layer 2, 0.35 ± 0.02, 0.41 ± 0.03, 0.48 ± 0.03, 0.55 ± 0.03, 0.63 ± 0.03 and 0.69 ± 0.03%.

4 Phytate estimated via NIRS.

5 HiPhorius 40,000 FYT/g, Novonesis A/S, Bagsvaerd, Denmark.

All nutrients met Bovans White recommendations (Hendrix, 2020), except for P. The analyzed total P in the feeds were 0.34 % ± 0.02, 0.41 % ± 0.03, 0.49 % ± 0.02, 0.55 % ± 0.03, 0.61 % ± 0.06, and 0.69 % ± 0.04, respectively. An indigestible marker (Celite Corp., Lompoc, CA) was added at 1 % in the feeds of the last experimental period (54–57 wks). The limestone used in the experimental feeds had a geometric mean particle size of 633 ± 17 μm, and its solubility was determined according to the method of Kim et al. (2019) at 43.35, 52.16, and 60.55 % after 5, 15, and 30 min of incubation, respectively.

### Hen performance measurements

Productive performance was evaluated at the end of each 28 d period (29, 33, 37, 41, 45, 49, 53, and 57 wks) and included body weight gain (BWG), daily FI, feed conversion ratio (FCR) per dozen eggs and per kg of eggs, egg weight, egg mass, egg production, and total eggs per hen. Eggs were collected four times a day (08:30 am, 11:00 am, 2:00 pm, and 4:00 pm). Ileal digesta was sampled from 15 hens per treatment after euthanasia by cervical dislocation on the last day of the trial. Digesta were gently flushed with distilled water from the intestinal segment extended from Meckel’s diverticulum to approximately 2 cm before the ileocecal junction. Samples were placed into plastic containers, immediately frozen in liquid nitrogen, and subsequently stored at −20°C until lyophilization (Christ Alpha 2-4 LD freeze dryer; Martin Christ Gefriertrocknungsanlagen GmbH, Osterode am Harz, Germany). Both feeds and lyophilized digesta were then ground to pass through a 0.5-mm screen (TE-631/2 knife mill; Tecnal, Piracicaba, SP, Brazil)

### Analysis and calculations

The following analyses were done with feeds and ileal digesta: DM after oven drying at 105°C (method 934.01; AOAC International, 2006); Ca and P (AOAC, 2019), and acid-insoluble ash (Choct and Annison, 1992; Vogtmann et al., 1975). Crude protein (N × 6.25) was measured by the combustion method (968.06; AOAC International, 2006), and GE was determined using a bomb calorimeter calibrated with benzoic acid as a standard (IKA C2000 calorimeter; IKA-Werke GmbH & Co. KG, Staufen, Germany). Phytase activity in the feeds was expressed in phytase yield units (FYT), defined as the activity that releases 1 µmol of inorganic phosphate from 5.0 mM sodium phytate per minute at pH 5.5 and 37°C (Engelen et al., 1994). Apparent ileal digestibility of Ca, P, GE, and CP, as well as IDE, were calculated using the equations described by Kong and Adeola (2014).

### Egg trait measurements

Twenty eggs per treatment were randomly collected during the last two days of each experimental period for egg quality evaluation. Eggshell, albumen, and yolk weights were recorded and expressed as a percentage of whole egg weight. Eggshells were washed with filtered water and dried at 105°C overnight before determining eggshell proportion. Eggshell thickness was measured three times at the equatorial region of each egg using a digital micrometer (Model IP65; Mitutoyo Corp., Kawasaki, Japan), and the average value was used for statistical analysis. Additionally, 20 eggs per treatment were used to determine eggshell breaking strength using a texture analyzer (Model TA.XT.plus Texture Analyzer; Texture Technologies Corp., Hamilton, MA) with a 75-mm (P/75) breaking probe as described by Molino et al. (2015).

### Statistical analysis

Data were tested for normality and homogeneity of variances using the Shapiro–Wilk and Levene tests, respectively (Levene, 1960; Shapiro and Wilk, 1965). When required, data were transformed using the arcsine square root transformation (z = asin(sqrt(y + 0.5))) (Ahrens et al., 1990). Analyses were performed using S.A.S. 9.4 (2013), and significance was declared at P ≤ 0.05.

Performance variables measured over time (BW, FI, FCR [kg/kg and kg/dozen], egg production [%], and egg quality traits) were analyzed using PROC MIXED with repeated measures, including phytase as a fixed qualitative factor, avP as a quantitative factor, period as a source of variation, and cage as the experimental unit and random effect. The covariance structure (AR(1)) was selected based on the Akaike information criterion (Littell et al., 1998). Variables not measured over time, including BWG, total egg production, and digestibility variables, were analyzed using PROC GLM. Linear and quadratic effects of avP were evaluated by regression analysis, with avP used as the primary response criterion and corresponding dP values presented for practical feed formulation purposes. Phytase avP equivalency was estimated by linear interpolation of the observed responses between adjacent dietary avP levels in hens not supplemented with phytase, using egg production and FCR as response criteria. When significant quadratic effects were detected, the avP concentration maximizing the response was estimated from the vertex of the equation (x = −b/2a). Mean separation was performed using Tukey’s test (Tukey, 1991).

## Results

Analyzed total P concentrations averaged 0.34 ± 0.02, 0.41 ± 0.03, 0.49 ± 0.02, 0.55 ± 0.03, 0.61 ± 0.06, and 0.69 ± 0.04 % for feeds formulated to 0.29, 0.35, 0.42, 0.49, 0.55, and 0.62 % total P, respectively. These values correspond to 0.09, 0.16, 0.23, 0.30, 0.37, and 0.44 % avP, and were respectively equivalent to 0.07, 0.12, 0.17, 0.22, 0.26, and 0.31 % dP.

Performance results are presented in Table 2. Significant phytase × avP interactions were observed for BWG (P = 0.0183), FCR (kg feed/kg egg mass; P = 0.0122), percent hen-day egg production (P = 0.0172) and number of total eggs/hen (P = 0.0002). Body weight gain increased linearly with avP, with a greater slope in the absence of phytase (Y = −10.59 + 601.56x; P < 0.0001) than with its supplementation (Y = 66.91 + 235.35x; P = 0.0215), corresponding to increases of 42 and 16 g, respectively, for each 0.07 percentage-point increase in avP. Feed conversion ratio (kg feed/kg egg mass) responded quadratically to avP in the absence of phytase (Y = 2.0807 − 1.8098x + 2.749x²; P < 0.0001), with an estimated optimum at 0.33 % avP whereas in phytase-supplemented feeds, FCR was affected linearly (Y = 1.8073 − 0.1685x; P = 0.0052). Overall, phytase improved FCR by 1.7 % (1.802 vs. 1.834; P = 0.0037). Percent hen-day egg production, in the absence of phytase, responded quadratically to avP (Y = 78.92 + 70.55x − 96.33x²; P = 0.0004), with a maximum response at 0.37 % avP whereas in phytase-supplemented feeds, egg production increased linearly as avP was higher (Y = 91.1930 + 6.7908x; P < 0.0001). Overall, phytase increased hen-day egg production by 2 percentage points (96.2 vs. 94.2 %; P < 0.0001).

**Table 2:** Cumulative performance of laying hens fed supplemental phytase and graded increases in dietary P from 26 to 57 wks of age.

Phytase, FYT/kg^1^	BWG, g/hen^3^	BW, kg	FI, g/hen/day	FCR, kg feed/kg egg mass	FCR, kg feed/dozen eggs	Hen-day egg production, %	Total eggs, eggs/hen
None	−23.7^b^	1.61^b^	111.6^b^	1.834^a^	1.352	94.2^b^	213^b^
4,000	37.8^a^	1.65^a^	113.5^a^	1.802^b^	1.341	96.2^a^	217^a^
AvP, %^2^							
0.09	−107.9^d^	1.59^b^	110.0^b^	1.890^a^	1.377^a^	91.8^c^	206^b^
0.16	8.11^c^	1.65^a^	112.8^a^	1.850^b^	1.367^ab^	94.2^b^	213^ab^
0.23	−9.46^b^	1.62^ab^	122.8^a^	1.796^c^	1.336^b^	95.9^ab^	215^ab^
0.30	52.4^a^	1.63^ab^	112.4^a^	1.780^c^	1.332^b^	95.9^ab^	216^a^
0.37	26.7^a^	1.62^ab^	113.5^a^	1.795^c^	1.335^b^	96.7^a^	218^a^
0.44	72.5^a^	1.67^a^	113.6^a^	1.795^c^	1.334^b^	96.8^a^	218^a^
SEM	0.01	0.003	0.20	0.004	0.002	0.15	0.2
*Probabilities*							
Phytase	0.0069	0.0147	0.0044	0.0037	0.1454	<.0001	0.0272
AvP linear	<.0001	0.0088	0.0014	<.0001	0.0001	<.0001	<.0001
AvP quadractic	0.4423	0.9089	0.2141	<.0001	0.0102	<.0001	0.0007
Phytase*AvP linear	0.0183	0.1978	0.0571	0.0466	0.1489	0.0002	0.0002
Phytase*AvP quadratic	0.4208	0.9178	0.4433	0.0122	0.1235	0.0172	0.2337

a-d Means with different letters in the same column indicate significant differences (P < 0.05) for main effects only.

1 HiPhorius 40,000 FYT/g, Novonesis A/S, Bagsvaerd, Denmark.

2 Analyzed P with and without phytase were: Layer 1, 0.34 ± 0.02, 0.41 ± 0.02, 0.49 ± 0.02, 0.55 ± 0.02, 0.62 ± 0.03 and 0.69 ± 0.04%; Layer 2, 0.35 ± 0.02, 0.41 ± 0.03, 0.48 ± 0.03, 0.55 ± 0.03, 0.63 ± 0.03 and 0.69 ± 0.03%.

3 BWG (0 FYT/kg): Y = −10.59 + 601.56x; BWG (4,000 FYT/kg): Y = 66.91 + 235.35x, BW: Y = 1.582 + 0.1344x, FI: Y = 109.86 + 7.3825x, FCR kg/kg (0 FYT/kg): Y = 2.0807 − 1.8098x + 2.749x²; optimal avP = 0.33%; FCR kg/kg (4,000 FYT/kg): Y = 1.8073−0.1685x; FCR kg/dozen eggs: Y = 1.4737 − 0.8238x + 1.2068x²; optimal avP = 0.34%; egg production (0 FYT/kg): Y = 78.92 + 70.55x − 96.33x²; optimal avP = 0.37%; egg production (4,000FYT/kg): Y = 91.1930+6.7908x; total egg production (0 FYT/kg): Y = 201.48 + 40.99x; total egg production (4,000 FYT/kg): Y = 212.01 + 16.37x; P < 0.0001.

The total eggs/hen produced during the experimental period increased linearly both without phytase (Y = 201.48 + 40.99x; P < 0.0001) as in phytase-supplemented hens (Y = 212.01 + 16.37x; P < 0.0001). Overall, hens supplemented with phytase produced approximately four more eggs per hen than those not supplemented (217 vs. 213 eggs/hen; P = 0.0272). Average daily FI (g/hen/day) was increased by phytase supplementation (113.5 with phytase vs. 111.6 without phytase; P = 0.0044) increasing linearly with dietary avP (P = 0.0014) (Y = 109.86 + 7.3825x; P = 0.0088), corresponding to an estimated increase of 0.52 g/hen per day for each 0.07 percentage-point increase in avP. Feed conversion ratio expressed as kg of feed per dozen eggs was not affected by phytase (P = 0.1454) but responded quadratically to avP (P = 0.0102) (Y = 1.4737 − 0.8238x + 1.2068x²; P = 0.0070), with an estimated optimum avP concentration of 0.34 % avP.

Egg trait results are presented in Table 3. No phytase × avP interactions were observed (P > 0.05); however, phytase affected all traits, except for albumen percentage, increasing egg weight (+0.5 g; P = 0.0032), eggshell proportion (+0.19 %; P < 0.0001), shell breaking strength (+0.17 kgf; P = 0.0022), and shell thickness (+6 µm; P = 0.0002), while decreasing yolk percentage (−0.3 %; P = 0.0007). Egg weight responded quadratically to avP (P = 0.0338) (Y = 59.38 + 14.14x − 23.42x²; P = 0.0348), with an estimated optimum at 0.30 % avP. Yolk percentage decreased linearly with avP (P = 0.0059) (Y = 28.227 − 1.4933x; P = 0.0061), whereas albumen percentage and shell breaking strength were unaffected (P > 0.05). Eggshell proportion (P = 0.0012) and shell thickness (P = 0.0071) increased linearly with avP (Y = 8.396 + 0.7215x; P = 0.0015; and Y = 358.34 + 30.16x; P = 0.0110, respectively), corresponding to increases of 0.05 % and 2.1 µm, respectively, for each 0.07 % increase in avP.

**Table 3:** Egg traits from laying hens fed supplemental phytase and graded increases in dietary P from 26 to 57 wks of age.

Phytase, FYT/kg^1^	Egg, g^3^	Yolk, %	Albumen, %	Shell, %	Breaking strength, kgf	Thickness, µm
None	61.4^b^	27.1^a^	63.9	8.94^b^	3.88^b^	378^b^
4,000	61.9^a^	26.8^b^	64.1	9.13^a^	4.05^a^	384^a^
AvP, %^2^						
0.09	60.9	27.2^ab^	63.9	8.95^b^	3.89	378^bc^
0.16	61.7	27.3^a^	63.7	8.99^b^	3.88	378^bc^
0.23	61.6	27.2^ab^	63.9	8.88^b^	3.90	375^c^
0.30	62.1	26.6^c^	64.4	9.08^a^	4.10	384^ab^
0.37	61.9	26.9^bc^	64.0	9.09^a^	4.06	385^a^
0.44	61.6	26.7^c^	64.1	9.21^a^	4.00	387^a^
SEM	0.09	0.05	0.05	0.024	0.029	0.8
*Probabilities*						
Phytase	0.0032	0.0007	0.2027	<.0001	0.0022	0.0002
AvP linear	0.1314	0.0059	0.2022	0.0012	0.1651	0.0071
AvP quadractic	0.0338	0.5499	0.4938	0.8683	0.3406	0.3580
Phytase*AvP linear	0.9804	0.9341	0.8541	0.7582	0.8967	0.4383
Phytase*AvP quadratic	0.6932	0.5770	0.8869	0.1076	0.8312	0.6863

a-c Means with different letters in the same column indicate significant differences (P < 0.05) for main effects only.

1 HiPhorius 40,000 FYT/g, Novonesis A/S, Bagsvaerd, Denmark.

2 Analyzed P with and without phytase were: Layer 1, 0.34 ± 0.02, 0.41 ± 0.02, 0.49 ± 0.02, 0.55 ± 0.02, 0.62 ± 0.03 and 0.69 ± 0.04%; Layer 2, 0.35 ± 0.02, 0.41 ± 0.03, 0.48 ± 0.03, 0.55 ± 0.03, 0.63 ± 0.03 and 0.69 ± 0.03%.

3 Egg weight: Y = 59.38 + 14.14x − 23.42x²; optimal avP = 0.30%; yolk percentage: Y = 28.227 − 1.4933x., eggshell percentage: Y = 8.396 + 0.7215x; eggshell thickness: Y = 358.34 + 30.16x.

Digestibility results obtained at the end of the trial are presented in Table 4. Significant phytase × avP interactions were observed for digestibility of DM (P = 0.0317) and CP (P = 0.0081) as well as for IDEC (P = 0.0152) and IDE (P = 0.0367). For DM digestibility, phytase-supplemented hens showed a quadratic response to avP (Y = 68.76 + 57.48x − 273.59x²; P = 0.0238) with an estimated maximum at 0.11 % avP, whereas no regression fit was observed in the absence of phytase for avP (P > 0.05). Crude protein digestibility was best fit to Y = 84.72 + 30.25x − 279.42x² (P = 0.0030) in phytase fed hens with an estimated maximum at 0.05 % avP, whereas no significant response to avP was observed in the absence of phytase (P > 0.05). In the absence of phytase, neither IDEC nor IDE was affected by dietary P (P > 0.05). In phytase-supplemented feeds, IDEC and IDE increased linearly with increasing P concentration and were fit to Y = 74.29 + 50.17x (P = 0.0006) and Y = 2960.35 + 2017.58x (P = 0.0005), respectively.

**Table 4:** Apparent ileal digestibility when laying hens were fed supplemental phytase and graded increases in dietary P on wk 57, dry matter basis^1^.

Phytase, FYT/kg^2^	DM, %^4^	IDEC, %	IDE, kcal/kg	CP, %	P, %	Ca, %
None	65.8	71.6	2,837	82.1	48.9^b^	77.2
4,000	68.8	74.3	2,960	84.7	72.5^a^	73.5
AvP, %^3^						
0.09	59.4	64.0	2,536	77.9	57.9	85.6^a^
0.16	65.2	73.3	2,873	80.9	68.9	82.2^ab^
0.23	61.2	66.5	2,664	82.2	60.8	74.9^b^
0.30	68.6	74.5	2,971	84.2	57.4	72.5^b^
0.37	72.9	77.0	3,072	84.2	64.1	77.1^ab^
0.44	68.3	72.2	2,916	81.6	61.3	71.9^b^
SEM	1.19	1.30	51.28	1.36	2.16	1.09
*Probabilities*						
Phytase	0.3789	0.4815	0.4143	0.2720	<.0001	0.2347
AvP linear	0.9039	0.8137	0.7528	0.4377	0.4994	0.0430
AvP quadractic	0.3495	0.7459	0.9316	0.4536	0.9937	0.7946
Phytase*AvP linear	0.0043	0.0152	0.0367	0.0064	0.4034	0.4368
Phytase*AvP quadratic	0.0317	0.1284	0.2608	0.0081	0.9966	0.2109

a-b Means with different letters in the same column indicate significant differences (P < 0.05) for main effects only.

1 DM, dry matter; IDCE, ileal digestible energy coefficient; IDE, ileal digestible energy.

2 HiPhorius 40,000 FYT/g, Novonesis A/S, Bagsvaerd, Denmark.

3 Analyzed P with and without phytase were: Layer 1, 0.34 ± 0.02, 0.41 ± 0.02, 0.49 ± 0.02, 0.55 ± 0.02, 0.62 ± 0.03 and 0.69 ± 0.04%; Layer 2, 0.35 ± 0.02, 0.41 ± 0.03, 0.48 ± 0.03, 0.55 ± 0.03, 0.63 ± 0.03 and 0.69 ± 0.03%.

4 DM digestibility (4,000 FYT/kg): Y = 68.76 + 57.48x − 273.59x²; optimal avP = 0.11%. IDEC (4,000 FYT/kg): Y = 74.29 + 50.17x. IDE (4,000 FYT/kg): Y = 2960.35 + 2017.58x. CP digestibility (4,000 FYT/kg): Y = 84.72 + 30.25x − 279.42x²; optimal avP = 0.05%. Ca digestibility: Y = 77.16 − 31.24x.

There was no interaction between phytase and avP for the digestibility of P (P > 0.05). However, this response was markedly improved by phytase supplementation (72.5 vs. 48.9 %; P < 0.0001) whereas dietary avP concentration had no significant effects (P > 0.05). As with P, there was no phytase × avP interaction or neither single effect of phytase on Ca digestibility (P = 0.2347); however, this decreased linearly as dietary avP increased (P = 0.0430) (Y = 77.16 − 31.24x; P = 0.0007) indicating a reduction of 2.2 % in Ca digestibility for each 0.07 % dietary increase in avP.

Phytase × AvP interaction was found for some responses and demonstrates that phytase partially mitigated the negative effects of severe P deficiency (Table 5). At the lowest AvP level (0.09 %), phytase improved BWG from −167.6 to −48.1 g/hen, increased hen-day egg production from 88.9 to 94.7 %, increased total egg production from 201 to 212 eggs/hen, and reduced FCR from 1.946 to 1.834 kg feed/kg egg mass. For nutrient digestibility variables, differences were observed only for the diet containing 0.09 % AvP supplemented with phytase, whereas no differences were detected among the remaining dietary P levels.

**Table 5:** Interaction between phytase supplementation and dietary P from 26 to 57 wks of age.

Phytase, FYT/kg^1^	AvP^2^	BWG, g/hen	FCR, kg feed/kg egg mass	Hen-day egg production, %	Total eggs, eggs/hen	IDDM, %^3^	IDEC, %	IDE, kcal/kg	CP, %
0	0.09	−167.6^c^	1.946^a^	88.9^c^	201^c^	69.3^a^	73.2^a^	2,803ª	85.5^a^
	0.16	−37.3^b^	1.860^ab^	93.1^b^	209^b^	69.3^a^	75.4^a^	2,903ª	83.1^a^
	0.23	−41.4^b^	1.797^b^	95.4^ab^	214^ab^	60.1^a^	67.5^a^	2,701ª	83.5^a^
	0.30	14.8abc	1.792^b^	95.2^ab^	214^ab^	67.6^a^	72.6^a^	2,886ª	82.2^a^
	0.37	−6.11abc	1.795^b^	96.3^a^	218^a^	68.3^a^	71.9^a^	2,855ª	80.9^a^
	0.44	95.5^a^	1.811^b^	96.4^a^	217^a^	69.1^a^	72.2^a^	2,884ª	83.3^a^
4,000	0.09	−48.1^b^	1.834^b^	94.7^ab^	212^b^	50.8^b^	56.1^b^	2,305^b^	71.4^b^
	0.16	53.5^ab^	1.840^b^	95.2^ab^	216^ab^	61.6ª	71.5ª	2,846ª	79.1ª
	0.23	22.5abc	1.795^b^	96.3ª	216^ab^	62.8ª	65.6ª	2,632ª	81.2ª
	0.30	90.0^a^	1.768^b^	96.6ª	218ª	69.5ª	76.2ª	3,045ª	85.8ª
	0.37	59.6^ab^	1.796^b^	97.1ª	219ª	76.9ª	81.5ª	3,259ª	87.1ª
	0.44	49.6^ab^	1.778^b^	97.1ª	218^a^	67.7ª	72.1ª	2,944^a^	80.1ª
SEM		31.88	0.018	0.66	5.7	10.74	12.19	484.8	7.81

a-c Means with different letters in the same column indicate significant differences (P < 0.05).

1 HiPhorius 40,000 FYT/g, Novonesis A/S, Bagsvaerd, Denmark.

2 Analyzed P with and without phytase were: Layer 1, 0.34 ± 0.02, 0.41 ± 0.02, 0.49 ± 0.02, 0.55 ± 0.02, 0.62 ± 0.03 and 0.69 ± 0.04%; Layer 2, 0.35 ± 0.02, 0.41 ± 0.03, 0.48 ± 0.03, 0.55 ± 0.03, 0.63 ± 0.03 and 0.69 ± 0.03 %. ^3^IDDM, IDCE, IDE, ileal digestible dry matter, energy coefficient and energy.

## Discussion

The findings of the present study demonstrated that the responses of laying hens to increasing dietary avP depended on phytase supplementation, as evidenced by the significant phytase × avP interactions observed for BWG, FCR and egg production. Rather than simply improving performance, phytase modified the magnitude of the response to dietary P, partially compensating for P deficiency at the lowest avP concentrations. This effect was particularly evident in hens fed 0.09 % avP, where phytase markedly improved BWG, egg production, and FCR, demonstrating its ability to alleviate the detrimental effects of severe P deficiency. Consequently, the differences between phytase-supplemented and unsupplemented hens progressively diminished as dietary avP increased. This type of response has been previously reported through the release of phytate-bound P and included an increase in FI and egg production (Liu et al., 2007; Rutherfurd et al., 2002; Teng et al., 2020).

The magnitude of the compensatory effect of supranutritional phytase can be illustrated by its estimated avP equivalency based on productive responses. Using egg production as the response criterion, hens fed without inorganic phosphate supplementation (0.09 % avP) but supplemented with phytase achieved a production rate comparable to that of hens fed approximately 0.21 % avP without phytase, corresponding to an avP equivalency of about 0.12 percentage units (approximately 0.09 % of dP). Likewise, based on FCR (kg feed/kg egg mass), phytase supplementation was equivalent to approximately 0.10 percentage units of avP (approximately 0.07 % of dP). These estimates demonstrate that phytase effectively compensated for dietary P deficiency under the most P-limiting conditions, whereas its relative contribution declined as dietary avP increased and P became less limited.

The reductions in FI, BW, and FCR observed under low P conditions are consistent with previous reports demonstrating that dietary P deficiency compromises performance in laying hens (Li et al., 2021; Teng et al., 2020; Yan et al., 2001). Although many of the mechanisms underlying reduced FI remain unclear, evidence from broiler studies suggests that P status may influence appetite through central and peripheral signaling pathways (Aderibigbe et al., 2022). Poorer FCR at lower avP concentrations may also reflect the greater challenge of maintaining Ca and P homeostasis under inadequate mineral supply (Lim et al., 2003). Hens rely heavily on medullary bone resorption to sustain eggshell formation, a process that becomes increasingly important when P availability is limited (Bar and Hurwitz, 1984; Nie et al., 2018; Sinclair-Black et al., 2023). By improving P and Ca availability, phytase may have reduced reliance on bone mineral mobilization, thereby supporting productive performance (Bello et al., 2020; Ren et al., 2023).

Although phytase induced only minor changes in internal egg components, its effects on eggshell quality were well pronounced. The slight reduction in yolk percentage observed with phytase added to the feeds likely reflects a proportional effect associated with increased egg and shell mass rather than a true alteration in yolk deposition, whereas albumen proportion remained unaffected. Hens fed the lowest dietary avP concentrations exhibited poorer eggshell quality, indicating that P supply was insufficient to sustain optimal shell formation. Because the eggshell contains only small amounts of P and is composed predominantly of Ca, this response was likely a consequence of the impaired bone mineral replenishment following the mobilization of Ca for eggshell synthesis. Under inadequate P supply, incomplete restoration of bone mineral reserves may reduce the availability of Ca for subsequent eggshell calcification, thereby compromising shell quality (Dacke et al., 1993; Li et al., 2018; Whitehead, 2004; Yan et al., 2023). The present findings are supported by previous reports demonstrating impaired eggshell quality in P-deficient feeds for laying hens (Englmaierová et al., 2015; Shao et al., 2025), although responses to dietary P deficiency have varied among studies (Jing et al., 2018; Pongmanee et al., 2020). The positive effects of phytase on shell thickness and breaking strength suggest enhanced release and utilization of Ca and P from phytate complexes, as previously reported by Gordon and Roland (1998). Similar improvements in shell quality and mineral retention have been reported with phytase supplemented from 1,000 to 3,000 FYT/kg, doses higher than usually added, (Eltahan et al., 2023; Lima et al., 2024), reinforcing the role of phytase in improving mineral availability for eggshell formation.

Dietary phytase increased ileal P digestibility by 23.6 %, confirming that the enzyme was functionally active under the conditions of the present study. Despite the limited main effects of phytase on some digestibility variables, the significant phytase × avP indicates that phytase efficacy depended on dietary P supply. In contrast, ileal Ca digestibility was influenced only by dietary avP levels, with no detectable response to phytase supplementation or phytase × avP interaction. The relatively wide Ca:P ratio adopted in the present study may have contributed to these responses by promoting the formation of insoluble Ca–phytate and Ca–phosphate complexes, which are known to reduce phytate solubility and depress P digestibility (Liu et al., 2013; Nahm, 2007; Selle et al., 2009; Tamim et al., 2004).

The responses observed for DM, CP, and energy utilization further suggest that phytase improved nutrient availability beyond P release, as has been previously shown (Cowieson et al., 2011; Dersjant-Li et al., 2015). Although Ca digestibility decreased linearly with dietary avP, it was not affected by phytase supplementation, a response also reported by Musilová et al. (2017). In contrast, Oliveira et al. (2018) and Walters et al. (2019) observed positive phytase effects on Ca digestibility, suggesting that responses depend on dietary characteristics such as Ca and P concentrations, phytate content, Ca ratio, and Ca source.

Collectively, the findings presented in this study indicate that phytase supplementation improved the efficiency of P utilization and improved the response sof laying hens fed proportionally reduced dietary avP. The variability in the dietary P concentrations that optimized all responses further highlights the difficulty of defining a single P requirement and supports the use of phytase as a practical tool to improve nutrient utilization and reduce the need for inorganic phosphate supplementation.

## Conclusion

The supranutritional phytase supplementation improved productive performance, eggshell quality over a 32 wk of egg production. Significant phytase × avP interactions demonstrated that phytase alleviated the effects of low dietary P supply and modified the response of hens that corresponded to higher dietary P. Feeds without phytase required approximately 0.33 to 0.37 % avP to maximize productive responses, whereas supplementation with 4,000 FYT/kg phytase provided an avP equivalency of 0.10 to 0.12 percentage units, indicating that dietary avP concentrations between 0.23 and 0.27 % may be sufficient to maintain productive performance while reducing the inclusion of inorganic phosphate in commercial layer feeds added with supranutritional phytase.

## Author contribution

**R. M. Horn**: conception, design of the work, analysis, interpretation of data, writing the manuscript draft and reviewing it critically; final approval of the version to be published; accountability for all aspects of the work. **S. L. Vieira**: conception, design of the work, acquisition, interpretation of data, writing the manuscript draft and reviewing it critically; final approval of the version to be published; accountability for all aspects of the work. **D. D. B. Maria**: conception, design of the work, analysis, interpretation of data, writing the manuscript draft and reviewing it critically; final approval of the version to be published; accountability for all aspects of the work. **Y. J. Olabarriaga**: Analysis, interpretation of data, writing the manuscript draft and reviewing it critically; final approval of the version to be published; accountability for all aspects of the work. **W. E. Altevogt**: Analysis, interpretation of data, writing the manuscript draft and reviewing it critically; final approval of the version to be published; accountability for all aspects of the work. **L. A. L. da Cunha**: Analysis, interpretation of data, reviewing the manuscript critically; final approval of the version to be published; accountability for all aspects of the work. **R. F. B. Leiskosky**: Analysis, interpretation of data, reviewing the manuscript critically; final approval of the version to be published; accountability for all aspects of the work.

## Disclosures

All authors declare that they have no conflict of interest or personal relationships that could have appeared to influence the work reported in this article.
